# Predictors and impact of trust on vaccine decisions in parents of 2-year-old children in Canada: findings from the 2017 Childhood National Immunization Coverage Survey (cNICS)

**DOI:** 10.1186/s12889-023-16705-5

**Published:** 2023-09-15

**Authors:** Schellenberg N, Dietrich Leurer M, Petrucka P, Crizzle AM

**Affiliations:** 1 School of Public Health, University of Saskatchewan, Saskatoon, SK Canada; 2 College of Nursing, University of Saskatchewan, Saskatoon, SK Canada

**Keywords:** Vaccine hesitancy, Acceptance, Canada, Preschool vaccines, Childhood vaccines, Knowledge, attitudes and beliefs (KAB), cNICS, Trust, Vaccine Information

## Abstract

Trust is known to be an important factor in vaccine decisions for parents of young children, but there has been a lack of Canadian data measuring the determinants and impact of trust. Using data from the 2017 Canadian Childhood National Immunization Coverage Survey (cNICS), this study analyzed the relationships between sources that parents trust for vaccine information and demographics, parental knowledge, attitudes, and beliefs (KAB) and vaccine decisions (refusal, delay or reluctance) in parents of 2-year-old children who had accepted at least one vaccine for their child (*n* = 6125). The findings show that 83% of parents trust doctors for vaccine information; 70–80% trust pharmacists, PMH, nurses and HC/PHAC; 34% trust family and 23% trust friends and CAM HCPs. However, parents found to have poor or moderate KAB were less likely to trust doctors, nurses, pharmacists, PMH and HC/PHAC. Parents were also less likely to trust the PMH or HC/PHAC if they had high school education or less or trade/college education, or were widowed, separated, or divorced. Parents who had never been reluctant to vaccinate their 2-year-old child were over 2 times more likely to trust doctors, nurses, pharmacists, PMH and HC/PHAC while parents who trusted family and friends were less likely to delay or refuse vaccines. There was also significant regional variation within Canada, with parents from Quebec most likely to trust doctors, nurses, pharmacists, friends, PMH and HC/PHAC. Parents from the Territories were less likely to trust doctors, nurses and pharmacists, but more likely to trust family. Parents were less likely to trust doctors if they were from the Prairies, and pharmacists if they were from BC, and parents from the Prairies and BC were less likely to trust HC/PHAC. Parents from Ontario were less likely to trust family or friends, but more likely to trust the PMH. Tailored vaccine campaigns are needed to account for educational, marital, and regional differences across Canada to improve vaccine uptake.

## Introduction

Vaccine decision making is a complex process for parents due to the increasing number of recommended vaccines, complexity of scientific data, and conflicting sources of information about vaccine safety and disease risk [1]. Parents who accept vaccines for their children may place some degree of trust in health care providers (HCPs) to interpret vaccine information appropriately, health care systems to deliver vaccines safely, governments to enact policies that are in citizens’ best interests, and/or pharmaceutical companies to produce safe and effective vaccines [1]. Trust can be defined as an intentional choice, a leap of faith, and a relationship where parents accept some vulnerability in vaccine decisions [1]. However, trust is not isolated to the individual parent but is influenced by external factors such as family, friends, and community or religious leaders, and generalised trust in members of society [1]. Furthermore, historical experiences of people who have been mistreated by government or who have had bad experiences with HCPs in the past continue to impact trust in health systems (e.g., The Tuskogee Syphilis Study for African Americans [2]; Sixties Scoop, forced sterilization, residential school legacy for Indigenous peoples in Canada) [3].

Prior studies showed that parents who distrust biomedical health systems, governments, and pharmaceutical companies, and relied on interpersonal social networks or CAM for health care advice were less likely to vaccinate their children whereas those who trusted family physicians, pharmacists, and governments for health care were more likely to vaccinate their children [4–11]. An example of how trust can impact vaccine uptake was seen in the United Kingdom (UK) following an article by Andrew Wakefield suggesting an increased risk of developing autism following the measles vaccine [12, 13]. As a result, parents began to distrust the government and physicians to provide unbiased information about this vaccine [14]. Measles vaccinations dropped and cases increased in the UK and throughout Europe with the largest impact in France where there were 24,000 new cases of measles that resulted in 1,000 Intensive Care Unit (ICU) admissions, 35 cases of encephalitis, and 15 deaths between 2008 and 2012 [13].

Canada's publicly funded health care system provides a standard set of vaccines for all residents with a primary series recommended for children aged seven and younger to protect against diphtheria, pertussis, poliomyelitis, tetanus, Haemophilus influenza B (Hib), invasive meningococcal C disease, invasive pneumococcal disease, rotavirus, measles, mumps, rubella, varicella, Hepatitis B (HB) and influenza [15]. Provincial governments are responsible to create their own vaccine guidelines based on funding and population priorities [15]. Therefore, while most children will have the opportunity to receive at least one dose of these vaccines by 2-years-old, there is variation across Canada for HB, measles and meningococcal vaccinations [15]. While prior studies show the impact that trust has on vaccine uptake (i.e., ‘complete’ or ‘incomplete’ vaccinations), there is limited data in Canada examining the impact of trust on specific vaccine decisions (e.g., refusal, delay or reluctance). For instance, one study found that parents who vaccinated their children trusted public health authorities, physicians, public health researchers, and pharmaceutical companies more often than those who did not [6]; another found that parents who were hesitant or whose children were not fully vaccinated were significantly less likely to trust vaccine information likely reflecting distrust in health institutions, governments, pharmaceutical companies and HCPs [16]. Other research showed that having regular access to a family doctor protects against incomplete vaccinations, implying trust in medical professionals [17, 18], while parents who chose CAM were three times more likely to delay [19], six times more likely to selectively vaccinate, and five times more likely to refuse their children's vaccinations [20]. Trust may also be related to parents’ KAB about vaccines, with some research suggesting that parents who accept vaccines are not always knowledgeable about them [5, 6] but may accept information if they trust the source [1, 21].

The purpose of this study was to describe and examine sources that parents of 2-year-old children in Canada trust for vaccine information and the impact on whether parents are reluctant to accept vaccines, or delay or refuse certain vaccines. The specific objectives were to determine: 1) where parents seek vaccine information; 2) sources that parents trust for vaccine information; 3) associations and predictors of sources that parents trust for vaccine information; and 4) if sources that parents trust for vaccine information predicts vaccine decisions (e.g., hesitancy, delay, or refusal).

## Methods

### Childhood National Immunization Coverage Survey (cNICS)

The cNICS has been administered every two years since 2011 by Statistics Canada and Public Health Agency of Canada (PHAC). The data includes information on vaccine acceptance, socio-demographic variables, geography, and KAB from parents of 2-, 7-, 14- and 17-year-old children collected through a telephone interview [22]. In 2017, the survey collected data from November 2017 to April 2018 with an overall sample size of 9072 [23]. The sampling frame was designed using the Canadian Child Benefit claims list since it includes approximately 96% of children in Canada, stratified by province, territory, sex and age [23]. The cNICS 2017 excluded children whose parents who do not collect Canadian Child Benefits, who live on First Nation reserves or are institutionalized [23]. The response rate in the 2-year-old cohort was 61.8% resulting in a sample size of 6519 [23].

### Protocol

The cNICS dataset was accessed through the Canadian Research Data Centre Network at the University of Saskatchewan. Approval to access the data was required by Statistics Canada as the data is not publicly available. Behavioural Research Ethics Board approval was not required (received an exemption) as the study was based on secondary data analyses with all data being de-identified by Statistics Canada.

### Inclusion/Exclusion criteria

Participants included in this study are survey respondents (hereafter referred to as parents) of children who were: 1) 2-years old; 2) recipients of at least one vaccine, and 3) born in Canada. The 2-year-old cohort would have had the opportunity to accept twelve vaccines at 2, 4, 6, 12 and 18 months old regardless of where they lived in Canada and was the only group in the 2017 cNICS that was asked all KAB questions. Parents were excluded from completing the KAB questions if they had refused all vaccines for their child, therefore only parents who had accepted at least one vaccine were included in this study. Parents were excluded if their 2-year-old child was born outside of Canada due to differing vaccine schedules and requirements based on their country of origin which may have created additional reasons for delaying or refusing a vaccine. These criteria excluded a total of 394 parents from the sample resulting in an unweighted sample size of 6125.

### Independent variables

Independent variables included in this study are parental age at child’s birth, sex, education, marital status, immigration to Canada, household income, number of household members, region of residence, Indigenous identity of child and KAB. For all variables, answers “I don’t know”, “refuse” or “other” were coded as missing and removed from analysis. As shown in Table 1, all independent variables were recoded into categorical data. The categories for each variable were adjusted to produce adequate cell sizes for analysis as required by Statistics Canada which was a minimum of 15 for weighted data.

**Table 1:** Summary of categorical variables

Variable	Categories
***Total Household Income***	$49,000 or less
	$49,100-$98,000
	$98,100-$151,900
	$152,000-$216,600
	$216,700 or more
***Responding Parent's Age (yrs.) at Child's Birth***	14 to 22
* > 54 yrs old coded as missing due to small cell sizes	23 to 30
	31 to 40
	41 to 54
***Household Size***	Two
* ≥ 7 coded as missing due to small cell sizes	Three or Four
	Five or Six
***Immigration***	Yes, in the last 10 years
	Yes, more than 10 years ago
	No, born in Canada
***Responding Parent's Marital Status***	Married/Common Law
	Widowed/Separated/Divorced
	Single/Never Married
***Responding Parent's Sex***	Male
	Female
***Responding Parent's Education Level***	High School or Less
	Trade/College Cert./Diploma
	University, Undergraduate
	University, Graduate
***Indigenous Identity of Child Reported by Parent***	Aboriginal Identity
*Terminology used in survey was 'Aboriginal Identity'	Non-Aboriginal Identity
***Region of Residence***	Atlantic (Newfoundland and Labrador, Nova Scotia, Prince Edward Island, New Brunswick)
	Quebec
	Ontario
	Prairies (Manitoba, Saskatchewan, Alberta)
	British Columbia
	Territories (Yukon, Northwest Territories, Nunavut)

Table listing variables and each sub-category within variables

KAB were assessed with 13 questions using a 4-item Likert scale (ranging from 1—strongly agree to 4—strongly disagree), as shown in Table 2. There were seven questions where “strongly agree” would indicate an accurate understanding of immunization and six where it would not. To simplify the analysis, the six questions that reflected an inaccurate understanding of immunization were reverse coded and combined with the seven questions that reflected accurate understanding of immunizations. Parents’ responses were added together which resulted in a total score ranging from 13–52. Cronbach’s alpha for the combined survey questions was 0.89 indicating strong internal consistency [24]. The total score was then split into three categories of vaccine knowledge: Accurate (13–25), in which parents would have answered ‘strongly agree’ (1) or ‘somewhat agree’ (2) to most questions; Moderate (26–35) in which parents would have answered ‘somewhat agree’ (2) or 'somewhat disagree’ (3); and Poor (36–52) which would include ‘somewhat disagree’ (3) or ‘strongly disagree’ (4) to most questions.

**Table 2:** Knowledge, Attitudes and Beliefs (KAB) regarding vaccination survey questions

Knowledge, Attitudes and Beliefs (KAB) Regarding Vaccination
In general, childhood vaccines are safe
In general, childhood vaccines are effective
In general, vaccines help to protect my child’s health
In general, a vaccine can*not* give you a serious case of the very same disease it was meant to prevent *(recoded)*
In general, the use of alternative practices (ie. homeopathy or chiropractic), can*not* eliminate the need for vaccination *(recoded)*
In general, a healthy lifestyle such as healthy nutrition and hygiene can*not* replace need for vaccination *(recoded)*
Having my child vaccinated helps to protect the health of others in my family
Having my child vaccinated helps to protect the health of others in my community
Children do *not* receive too many vaccines at the same visit *(recoded)*
Children do *not* receive too many vaccines overall *(recoded)*
It is *not* better for children to develop immunity from natural infections rather than from vaccines *(recoded)*
Delaying child vaccines causes risks to their health
Unvaccinated children are at higher risk of getting some serious diseases

Table with list of cNICS survey questions used to assess parents' vaccine knowledge, attitudes and beliefs

Previous vaccine decisions were included as dichotomous variables based on parents’ yes or no responses to three separate questions: 1) Have you ever refused a vaccine for your child? 2) Have you ever been reluctant to accept a vaccine for your child, excluding vaccines that have been refused? and 3) Have you ever delayed a vaccine for your child? These questions were not mutually exclusive. Responses “I don’t know” and “refused” were coded as missing and removed from analysis consistent with prior studies [19, 25].

### Outcome measures

The primary outcome variable is whether parents trust a specific source for vaccine information. The variable “who do you trust for vaccine information” assessed the level of trust in sources of information including doctors, nurses, pharmacists, CAM HCPs (naturopaths, chiropractors, etc.), family, friends, the Provincial Ministry of Health (PMH), and the Health Canada/Public Health Agency of Canada (HC/PHAC) on a 4-point Likert scale (1-really trust, 2- trust, 3- somewhat trust, 4- do not trust at all). The responses from the Likert scale were combined to create a binomial categorical response for *Yes* (1 and 2) and *No* (3 and 4); “don’t know” or “refuse to answer” were coded as missing data and removed from the analysis.

### Statistical analysis

The data used for analysis were weighted by Statistics Canada for non-response and stratification, and additional weights required for KAB analysis were applied resulting in a weighted sample size of 350,000. All data was bootstrapped with 1000 replications [26] to reflect the Canadian population. Independent and dependent variables were described using frequencies (percent). Chi Square tests were used to examine the association between independent and dependent variables and are reported as a design-based *F* statistic using a second order Rao and Scott correction due to the complex survey design [26]. Binomial Logistic Regression was performed using McFadden’s ${R}^{2}$ and Bayesian Information Criterion (BIC) for variable inclusion and goodness of fit [27, 28]. All statistical analysis was done using STATA 15.1 [29].

## Results

### Sample description

As shown in Table 3, of the parents of 2-year-old children born in Canada who had received at least one vaccine, 82.6% were from Ontario, Quebec and the Prairies with 30.6% being immigrants. Over 96% of parents had at least three members living in the household and 86.3% reported being married/common law. 53.8% of parents had a high school diploma or less, or college diploma, while more than a quarter had an income of $49,000 or less. Most parents were female (89.6%); only 5% reported being Indigenous. Based on the responses, 82.1% of parents had an accurate understanding, 15.8% had a moderate understanding, and 2.1% had a poor understanding of vaccines.

**Table 3:** Sample description of responding parent, including vaccine decisions (*n* = 350,000^a^)

Variable	%
***Region of Residence***	
Atlantic	5.7
Quebec	22.7
Ontario	37.8
Prairies	22.1
British Columbia	11.2
Territories	0.5
***Total Household Income***	
$49,000 or less	25.9
$49,100—$98,000	33.4
$98,100—$151,900	27.2
$152,000—$216,500	9.5
$216,700 or more	4.0
***Household Size***	
Two	3.2
Three or Four	66.0
Five or Six	30.9
***Immigration History***	
Yes, in the Last 10 Years	15.0
Yes, more than 10 Years Ago	15.6
No, born in Canada	69.4
***Age When Child Was Born***	
14 to 22	6.2
23 to 30	41.5
31 to 40	49.0
41 to 54	3.3
***Marital Status***	
Married/Common Law	86.3
Separated/Divorced/Widowed	4.5
Single/Never Married	9.2
***Sex***	
Male	10.4
Female	89.6
***Education Level***	
High School Diploma/Equivalent or Less	21.0
Trade/College Certificate or Diploma	32.8
University Undergraduate Level	32.2
University Certificate/Diploma/Degree Above Bachelor's Level	13.9
***Aboriginal Identity of Child***	
Aboriginal Identity	5.0
Non-Aboriginal Identity	95.0
***Level of Vaccine KAB***	
Accurate Understanding	82.1
Moderate Understanding	15.8
Poor Understanding	2.1
***Ever Refused Vaccine(s)***	
Refused Sometime	16.8
Never Refused	83.2
***Ever Delayed Vaccine(s)***	
Delayed sometime	13.1
Never Delayed	86.9
***Ever Reluctant to Accept Vaccine(s)***	
Reluctant Sometime	12.9
Never Reluctant	87.1

Data are presented using proportions

Table representing description of bootstrapped sample of responding parents of two-year old children, displayed using proportions

^a^All data are weighted and bootstrapped to obtain estimates at the Canadian population level

### Trusted sources of information

Parents stated they trusted doctors, HC/PHAC, and nurses the most as sources of vaccine information and reported that they most often sought vaccine information from those same three sources (see Table 4). Friends and CAM HCPs were trusted the least. Parents reported looking for vaccine information from social media, other sources or CAM HCPs 10.1% or less of the time while family, friends and pharmacists were sources of information 18.5–24.8% of the time.

**Table 4:** Trusted sources of vaccine information for responding parent (*n* = 350,000^a^)

	***Sources Parents Trust***	***Where do Parents Get Info?***
*Variable*	%	%
Doctors	83.4	82.2
Nurses	77.5	51.1
Pharmacists	70.6	24.2
CAM HCPs	23.5	7.8
Family	34.4	24.8
Friends	23.4	18.5
Provincial Ministry of Health	75.8	35.7
Health Canada/PHAC	80.9	40.8
Social Media	^**^	10.1
Does Not Seek Information	^**^	2.0
Other Sources	^**^	7.5

Data are presented using proportions

Table representing sources that parents trust for vaccine information and where they get information, displayed as proportions

^a^All data are weighted and bootstrapped to obtain estimates at the Canadian population level

^**^These sources were not included in the trust assessment

### Associations and predictors of sources that parents trust

Associations and predictors of sources that parents of 2-year-old children born in Canada who have received at least one vaccine trust for vaccine information and demographic variables, KAB and previous vaccine decisions are shown in for doctors, pharmacists, nurses and CAM HCPs in Table 5, and for family, friends, the provincial ministry of health and Health Canada/PHAC in Table 6, respectively. Tables 7, 8, 9, and 10 shows the predictors of trust for doctors and nurses, pharmacists and CAM HCPs, family and friends, and provincial health ministries and Health Canada/PHAC, respectively.

**Table 5:** Associations between demographics and sources that responding parents trust for vaccine information (*n* = 350,000^a^)

**A**								
	***Doctors***		***Nurses***		***Pharmacists***		***Alt. HCPs***	
	Yes (%)	p**	Yes (%)	*p*	Yes (%)	*p*	Yes (%)	*p*
***Total Household Income***		** < 0.001**		** < 0.001**		** < 0.001**		** < 0.001**
$49,000 or less	77.23		66.70		63.63		32.74	
$49,100-$98,000	82.88		79.55		70.38		25.50	
$98,100-$151,900	88.07		83.54		75.87		17.55	
$152,000-$216,600	85.94		79.27		74.13		16.20	
$216,700 or more	90.01		84.39		79.04		9.34	
***Age (yrs.) at Child's Birth***		**0.003**		**0.014**		0.140		**0.011**
14 to 22	73.60		68.05		64.22		32.67	
23 to 30	82.79		78.24		70.87		24.99	
31 to 40	85.27		78.88		72.42		21.18	
41 to 54	84.83		70.13		65.14		21.37	
***Household Size***		** < 0.001**		** < 0.001**		**0.047**		**0.045**
Two	71.79		62.88		64.48		35.28	
Three or Four	85.40		80.26		72.39		23.51	
Five or Six	80.54		73.97		67.91		21.95	
***Immigration History***		0.148		** < 0.001**		** < 0.001**		**0.002**
Yes, within the last 10 years	86.75		76.33		64.08		31.04	
Yes, more than 10 years ago	81.49		67.64		61.39		21.09	
No, born in Canada	83.19		80.54		74.76		22.35	
***Marital Status***		** < 0.001**		** < 0.001**		**0.047**		0.052
Married/Common Law	85		79.41		71.73		22.9	
Widowed/Separated/Divorced	81.26		72.47		69.48		22.12	
Single/Never Married	69.90		62.11		64.21		30.26	
***Sex***		0.684		0.663		0.095		0.163
Male	84.28		76.39		66.11		20.04	
Female	83.31		77.61		71.39		23.92	
***Education Level***		** < 0.001**		** < 0.001**		**0.008**		** < 0.001**
High School or Less	77.66		71.62		67.73		27.39	
Trade/College Certificate or Diploma	81.02		75.63		68.02		27.78	
University, Undergraduate level	87.28		82.26		75.12		20.45	
University, Graduate level	89.16		80.01		72.3		14.92	
***Aboriginal Identity of Child***		**0.001**		**0.008**		0.069		**0.007**
Aboriginal Identity	74.20		69.18		64.96		32.15	
Non-Aboriginal Identity	83.81		78.02		71.34		22.60	
***Region of Residence***		** < 0.001**		** < 0.001**		** < 0.001**		**0.046**
Atlantic	83.38		80.81		77		23.94	
Quebec	87.67		85.32		82.86		27.24	
Ontario	84.53		73.41		64.96		21.23	
Prairies	79.36		77.28		70.29		24.56	
British Columbia	79.69		74.68		64.70		21.2	
Territories	68.64		66.51		62.59		30.68	
***Level of Vaccine KAB***		** < 0.001**		** < 0.001**		** < 0.001**		** < 0.001**
Accurate Understanding	90.30		85.58		77.18		18.26	
Moderate Understanding	69.77		63.81		61.51		37.68	
Poor Understanding	21.41		18.52		27.25		53.17	
***Ever Decided to Delay a Vaccine(s)***		** < 0.001**		** < 0.001**		** < 0.001**		0.825
Delayed Sometime	73.88		68.18		60.47		24.06	
Never Delayed	84.94		78.93		72.44		23.46	
***Ever Refused Vaccine(s)***		** < 0.001**		**0.001**		**0.045**		0.054
Refused Sometime	73.67		71.84		66.97		27.11	
Never Refused	85.36		78.75		71.72		22.67	
***Ever Reluctant to Accept Vaccine(s)***		** < 0.001**		** < 0.001**		** < 0.001**		0.169
Reluctant Sometime	63.04		62.13		56.79		26.44	
Never Reluctant	86.44		79.75		72.93		22.95	
B								
	***Family***		***Friends***		***Prov. MoH***		***HC/PHAC***	
	Yes (%)	p**	Yes (%)	*p*	Yes (%)	*p*	Yes (%)	*p*
***Total Household Income***		** < 0.001**		**0.020**		**0.020**		**0.001**
$49,000 or less	42.70		27.63		74.08		77.33	
$49,100-$98,000	35.14		23.27		72.93		78.74	
$98,100-$151,900	29.85		22.01		79.64		85.43	
$152,000-$216,600	25.85		17.83		76.95		82.85	
$216,700 or more	26.81		20.26		80.46		86.35	
***Age (yrs.) at Child's Birth***		0.354		0.445		0.590		0.613
14 to 22	34.27		18.56		72.34		77.4	
23 to 30	33.77		24.47		76.00		81.56	
31 to 40	34.27		23.23		76.61		81.33	
41 to 54	44.01		21.64		71.9		77.58	
***Household Size***		0.571		0.173		**0.016**		**0.010**
Two	40.15		23.07		68.46		74.92	
Three or Four	34.25		24.76		77.57		82.9	
Five or Six	34.39		21.27		72.98		78.21	
***Immigration History***		**0.001**		**0.034**		**0.012**		**0.015**
Yes, within the last 10 years	42.62		28.36		82.56		86.26	
Yes, more than 10 years ago	36.01		20.13		74.63		76.85	
No, born in Canada	31.94		23.12		74.63		80.72	
***Marital Status***		0.06		0.583		**0.004**		**0.008**
Married/Common Law	33.60		23.09		76.87		81.88	
Widowed/Separated/Divorced	41.52		25.60		67.14		75.19	
Single/Never Married	39.37		25.86		69.83		74.96	
***Sex***		0.615		0.828		0.338		0.405
Male	35.83		23.98		78.19		83.05	
Female	34.25		23.35		75.49		80.68	
***Education Level***		**0.004**		0.348		** < 0.001**		** < 0.001**
High School or Less	40.10		22.79		70.32		75.29	
Trade/College Certificate or Diploma	35.47		25.19		70.68		76.5	
University, Undergraduate level	30.77		21.49		81.99		85.98	
University, Graduate level	30.83		24.49		82.48		88.48	
***Aboriginal Identity of Child***		0.512		0.704		**0.006**		**0.013**
Aboriginal Identity	36.26		22.06		66.90		73.19	
Non-Aboriginal Identity	33.83		23.32		76.25		81.33	
***Region of Residence***		**0.003**		** < 0.001**		**0.023**		** < 0.001**
Atlantic	34.94		23.79		72.78		82.32	
Quebec	39.48		32.72		78.45		86.44	
Ontario	30.74		18.28		77.31		80.11	
Prairies	35.89		22.6		72.88		78.88	
British Columbia	32.88		23.10		73.00		76.5	
Territories	49.18		33.94		59.79		69.31	
***Level of Vaccine KAB***		0.063		0.270		** < 0.001**		** < 0.001**
Accurate Understanding	31.35		21.87		82.84		88.44	
Moderate Understanding	38.28		25.66		59.85		64.09	
Poor Understanding	28.11		27.12		15.67		22.45	
***Ever Decided to Delay a Vaccine(s)***		**0.002**		**0.010**		** < 0.001**		** < 0.001**
Delayed Sometime	26.8		17.44		65.48		69.34	
Never Delayed	35.55		24.34		77.37		82.69	
***Ever Refused Vaccine(s)***		**0.001**		0.447		**0.001**		** < 0.001**
Refused Sometime	27.19		22.00		69.21		74.06	
Never Refused	35.78		23.75		77.05		82.27	
***Ever Reluctant to Accept Vaccine(s)***		0.088		0.050		** < 0.001**		** < 0.001**
Reluctant Sometime	30.21		18.90		54.42		60.89	
Never Reluctant	35.05		24.11		78.94		83.87	

Data are presented using proportions

Table presenting associations of sources that parents trust for vaccine information using bootstrapped data; results are displayed as proportions and p-value for Chi Square

^a^All data are weighted and bootstrapped to obtain estimates at the Canadian population level

^**^results with *p*-value < 0.05 are bold

**Table 6:** Predictors of sources that responding parents trust for vaccine information

A						
	***Doctors***			***Nurses***		
	*n* = 290,000, R2 = 0.151			*n* = 270,000, R2 = 0.130		
	SE	p*	OR (95% CI)	SE	*p*	OR (95% CI)
***Total Household Income***						
$49,000 or less	0.21	0.103	0.52 (0.23—1.14)	0.21	0.125	0.57 (0.28—1.17)
$49,100-$98,000	0.18	0.053	0.49 (0.24—1.01)	0.28	0.592	0.83 (0.43—1.62)
$98,100-$151,900	0.26	0.321	0.69 (0.34—1.43)	0.32	0.841	0.94 (0.48—1.81)
$152,000-$216,600	0.25	0.243	0.63 (0.29—1.37)	0.32	0.732	0.89 (0.44—1.78)
$216,700 and more	ref			ref		
***Parent's Age (yrs.) at Child's Birth***						
14 to 22	0.36	0.905	1.04 (0.53—2.06)	0.34	0.994	1.00 (0.52—1.93)
23 to 30	ref			ref		
31 to 40	0.19	0.427	1.14 (0.82—1.58)	0.14	0.763	0.96 (0.72—1.27)
41 to 54	0.55	0.739	1.17 (0.47—2.93)	0.35	0.654	0.83 (0.36—1.90)
***Household Size***						
Two	0.46	0.903	0.94 (0.36—2.45)	0.51	0.836	1.10 (0.44—2.75)
Three or Four	ref			ref		
Five or Six	0.14	0.295	0.84 (0.61—1.16)	0.13	0.182	0.81 (0.59—1.10)
***Immigration***						
Yes, in the last 10 years	0.28	0.810	1.07 (0.64—1.79)	0.17	0.182	0.74 (0.48—1.15)
Yes, more than 10 years ago	0.19	0.348	0.80 (0.50—1.28)	0.11	**0.003**	0.54 (0.36—0.82)
No, born in Canada	ref			ref		
***Parent's Marital Status***						
Married/Common-Law	ref			ref		
Widowed/Separated/Divorced	0.31	0.599	0.82 (0.39—1.72)	0.39	0.896	0.95 (0.43—2.11)
Single/Never Married	0.23	0.352	0.75 (0.41—1.38)	0.19	0.133	0.63 (0.34—1.15)
***Parent's Sex***						
Male	^**^			^**^		
Female						
***Parent's Education Level***						
High School or Less	0.22	0.560	0.86 (0.53—1.41)	0.19	0.365	0.81 (0.51—1.28)
Trade/College Certificate or Diploma	0.20	0.886	1.03 (0.70—1.52)	0.17	0.481	0.88 (0.61—1.27)
University, Undergraduate level	ref			ref		
University, Graduate level	0.28	0.834	1.06 (0.63—1.78)	0.18	0.410	0.84 (0.55—1.28)
***Aboriginal Identity of Child***						
Aboriginal Identity	0.39	0.788	1.10 (0.55—2.19)	0.26	0.571	0.84 (0.46—1.53)
Non-Aboriginal Identity	ref			ref		
***Region of Residence***						
Atlantic	ref			ref		
Quebec	0.47	**0.002**	2.02 (1.28—3.17)	0.41	**0.013**	1.77 (1.13—2.79)
Ontario	0.25	0.541	1.14 (0.75—1.74)	0.14	0.260	0.82 (0.59—1.16)
Prairies	0.11	**0.027**	0.72 (0.54—0.96)	0.11	0.118	0.80 (0.61—1.06)
British Columbia	0.13	0.071	0.73 (0.51—1.03)	0.15	0.332	0.84 (0.60—1.19)
Territories	0.10	**0.001**	0.46 (0.29—0.71)	0.12	**0.006**	0.57 (0.38—0.85)
***Parent Level of Vaccine KAB***						
Accurate Understanding	ref			ref		
Moderate Understanding	0.05	**0.000**	0.27 (0.19—0.39)	0.06	**0.000**	0.33 (0.23—0.46)
Poor Understanding	0.03	**0.000**	0.05 (0.02—0.15)	0.03	**0.000**	0.06 (0.02—0.16)
***Ever Decided to Delay a Vaccine(s)***						
Delayed Sometime	ref			ref		
Never Delayed	0.26	0.542	1.15 (0.73—1.80)	0.26	0.226	1.28 (0.86—1.90)
***Ever Refused Vaccine(s)***						
Refused Sometime	ref			ref		
Never Refused	0.33	**0.015**	1.63 (1.10—2.42)	0.22	0.246	1.23 (0.87—1.75)
***Ever Been Reluctant to Accept Vaccine(s)***						
Reluctant Sometime	ref			ref		
Never Reluctant	0.54	**0.000**	2.69 (1.81—3.99)	0.40	**0.000**	2.03 (1.38—3.00)
B						
	***Pharmacists***			***Alt. Health Providers***		
	n = 250,000, R2 = 0.0932			n = 79,000, R2 = 0.0684		
	SE	p*	OR (95% CI)	SE	*p*	OR (95% CI)
***Total Household Income***						
$49,000 or less	0.22	0.237	0.69 (0.37—1.28)	0.93	**0.006**	2.64 (1.32—5.27)
$49,100-$98,000	0.24	0.494	0.82 (0.47—1.44)	0.71	**0.016**	2.18 (1.15—4.12)
$98,100-$151,900	0.26	0.691	0.89 (0.51—1.57)	0.52	0.096	1.68 (0.91—3.09)
$152,000-$216,600	0.31	0.991	1.00 (0.54—1.83)	0.68	**0.043**	2.00 (1.02—3.91)
$216,700 and more	ref			ref		
***Parent's Age (yrs.) at Child's Birth***						
14 to 22	0.28	0.981	0.99 (0.57—1.72)	0.27	0.794	0.93 (0.52—1.65)
23 to 30	ref			ref		
31 to 40	0.15	0.250	1.16 (0.90—1.48)	0.13	0.983	1.00 (0.77—1.29)
41 to 54	0.50	0.536	1.27 (0.59—2.75)	0.42	0.792	1.11 (0.52—2.33)
***Household Size***						
Two	0.59	0.574	1.29 (0.53—3.17)	0.64	0.375	1.47 (0.63—3.47)
Three or Four	ref			ref		
Five or Six	0.13	0.850	0.98 (0.75—1.27)	0.13	0.451	0.90 (0.68—1.19)
***Immigration***						
Yes, in the last 10 years	0.11	**0.002**	0.55 (0.38—0.80)	0.27	0.098	1.39 (0.94—2.05)
Yes, more than 10 years ago	0.09	**0.000**	0.47 (0.33—0.68)	0.17	0.315	0.81 (0.53—1.22)
No, born in Canada	ref			ref		
***Parent's Marital Status***						
Married/Common-Law	ref			ref		
Widowed/Separated/Divorced	0.45	0.435	1.31 (0.67—2.56)	0.18	0.054	0.50 (0.25—1.01)
Single/Never Married	0.32	0.918	1.03 (0.57—1.89)	0.28	0.855	1.05 (0.62—1.78)
***Parent's Sex***						
Male	ref			ref		
Female	0.27	0.198	1.30 (0.87—1.94)	0.40	**0.018**	1.73 (1.10—2.71)
***Parent's Education Level***						
High School or Less	0.14	0.159	0.77 (0.54—1.11)	0.25	0.237	1.27 (0.86—1.88)
Trade/College Certificate or Diploma	0.10	**0.020**	0.71 (0.54—0.95)	0.23	**0.002**	1.58 (1.18—2.11)
University, Undergraduate level	ref			ref		
University, Graduate level	0.14	0.184	0.79 (0.55—1.12)	0.18	0.303	0.80 (0.52—1.23)
***Aboriginal Identity of Child***						
Aboriginal Identity	0.23	0.541	0.85 (0.50—1.44)	0.30	0.681	1.12 (0.66—1.88)
Non-Aboriginal Identity	ref			ref		
***Region of Residence***						
Atlantic	ref			ref		
Quebec	0.36	**0.000**	2.00 (1.41—2.86)	0.18	0.275	1.18 (0.88—1.60)
Ontario	0.13	0.090	0.76 (0.55—1.05)	0.14	0.201	0.80 (0.56—1.13)
Prairies	0.12	0.160	0.82 (0.63—1.08)	0.14	0.742	1.04 (0.81—1.34)
British Columbia	0.11	**0.006**	0.63 (0.45—0.88)	0.16	0.801	0.96 (0.69—1.33)
Territories	0.13	**0.022**	0.63 (0.43—0.94)	0.22	0.830	1.05 (0.70—1.57)
***Parent Level of Vaccine KAB***						
Accurate Understanding	Ref			ref		
Moderate Understanding	0.09	**0.000**	0.55 (0.39—0.76)	0.40	**0.000**	2.40 (1.73—3.34)
Poor Understanding	0.09	**0.000**	0.18 (0.07—0.47)	2.38	**0.000**	5.70 (2.51—12.95)
***Ever Decided to Delay a Vaccine(s)***						
Delayed Sometime	ref			ref		
Never Delayed	0.31	**0.002**	1.72 (1.21—2.44)	0.22	0.665	1.09 (0.74—1.61)
***Ever Refused Vaccine(s)***						
Refused Sometime	ref			ref		
Never Refused	0.17	0.778	1.05 (0.76—1.44)	0.15	0.707	0.94 (0.69—1.28)
***Ever Been Reluctant to Accept Vaccine(s)***						
Reluctant Sometime	ref			ref		
Never Reluctant	0.36	**0.000**	2.01 (1.41—2.86)	0.22	0.267	1.22 (0.86—1.73)
C						
	***Family***			***Friends***		
	*n* = 120,000, R2 = 0.0347			*n* = 82,000, R2 = 0.0404		
	SE	p*	OR (95% CI)	SE	*p*	OR (95% CI)
***Total Household Income***						
$49,000 or less	0.51	**0.028**	1.84 (1.07—3.15)	0.50	0.105	1.64 (0.90—2.97)
$49,100-$98,000	0.36	0.203	1.40 (0.84—2.33)	0.38	0.385	1.29 (0.73—2.29)
$98,100-$151,900	0.31	0.549	1.17 (0.70—1.96)	0.31	0.650	1.13 (0.66—1.95)
$152,000-$216,600	0.30	0.849	1.06 (0.61—1.83)	0.31	0.981	1.01 (0.55—1.85)
$216,700 and more	ref			ref		
***Parent's Age (yrs.) at Child's Birth***						
14 to 22	0.19	0.267	0.76 (0.46—1.24)	0.20	0.151	0.65 (0.36—1.17)
23 to 30	ref			ref		
31 to 40	0.15	**0.020**	1.31 (1.04—1.65)	0.14	0.334	1.13 (0.88—1.44)
41 to 54	0.81	**0.006**	2.47 (1.30—4.69)	0.42	0.767	1.12 (0.53—2.35)
***Household Size***						
Two	0.24	0.256	0.67 (0.33—1.34)	0.30	0.585	0.82 (0.39—1.69)
Three or Four	ref			ref		
Five or Six	0.12	0.792	0.97 (0.77—1.22)	0.11	0.315	0.88 (0.68—1.13)
***Immigration***						
Yes, in the last 10 years	0.22	0.122	1.30 (0.93—1.81)	0.20	0.606	1.10 (0.77—1.57)
Yes, more than 10 years ago	0.16	0.742	0.95 (0.68—1.32)	0.14	**0.044**	0.65 (0.42—0.99)
No, born in Canada	ref			ref		
***Parent's Marital Status***						
Married/Common-Law	ref			ref		
Widowed/Separated/Divorced	0.37	0.655	1.15 (0.62—2.16)	0.40	0.413	1.29 (0.70—2.36)
Single/Never Married	0.28	0.468	1.19 (0.75—1.89)	0.30	0.531	1.17 (0.71—1.94)
***Parent's Sex***						
Male	ref			ref		
Female	0.21	0.607	1.10 (0.76—1.59)	0.19	0.620	0.90 (0.59—1.37)
***Parent's Education Level***						
High School or Less	0.24	0.054	1.39 (0.99—1.94)	0.17	0.669	0.92 (0.64—1.33)
Trade/College Certificate or Diploma	0.17	**0.039**	1.31 (1.01—1.69)	0.17	0.144	1.23 (0.93—1.61)
University, Undergraduate level	ref			ref		
University, Graduate level	0.15	0.560	0.91 (0.66—1.25)	0.18	0.874	0.97 (0.68—1.38)
***Aboriginal Identity of Child***						
Aboriginal Identity	0.19	0.340	0.80 (0.50—1.27)	0.21	0.322	0.76 (0.44—1.32)
Non-Aboriginal Identity	ref			ref		
***Region of Residence***						
Atlantic	ref			ref		
Quebec	0.16	0.131	1.23 (0.94—1.59)	0.22	**0.000**	1.63 (1.26—2.12)
Ontario	0.11	**0.025**	0.72 (0.54—0.96)	0.11	**0.006**	0.64 (0.46—0.88)
Prairies	0.11	0.840	0.98 (0.79—1.21)	0.11	0.459	0.92 (0.73—1.16)
British Columbia	0.14	0.675	0.94 (0.71—1.25)	0.16	0.742	1.05 (0.79—1.41)
Territories	0.23	**0.016**	1.46 (1.07—1.99)	0.24	0.079	1.36 (0.97—1.91)
***Parent Level of Vaccine KAB***						
Accurate Understanding	ref			ref		
Moderate Understanding	0.21	0.208	1.24 (0.89—1.74)	0.19	0.559	1.11 (0.79—1.54)
Poor Understanding	0.43	0.928	1.04 (0.46—2.34)	0.80	0.138	1.88 (0.82—4.33)
***Ever Decided to Delay a Vaccine(s)***						
Delayed Sometime	ref			ref		
Never Delayed	0.29	**0.009**	1.61 (1.13—2.30)	0.32	**0.032**	1.56 (1.04—2.33)
***Ever Refused Vaccine(s)***						
Refused Sometime	ref			ref		
Never Refused	0.23	**0.022**	1.43 (1.05—1.95)	0.19	0.323	1.17 (0.85—1.62)
***Ever Been Reluctant to Accept Vaccine(s)***						
Reluctant Sometime	ref			ref		
Never Reluctant	0.20	0.291	1.20 (0.86—1.67)	0.30	0.058	1.48 (0.99—2.21)
D						
	***Prov. Min. of Health***			***Health Canada/PHAC***		
	*n* = 260,000, R2 = 0.116			*n* = 280,000, R2 = 0.145		
	SE	p*	OR (95% CI)	SE	*p*	OR (95% CI)
***Total Household Income***						
$49,000 or less	0.39	0.726	1.13 (0.57—2.23)	0.35	0.670	0.84 (0.37—1.91)
$49,100-$98,000	0.25	0.444	0.78 (0.42—1.46)	0.27	0.359	0.70 (0.33—1.50)
$98,100-$151,900	0.27	0.646	0.87 (0.47—1.60)	0.33	0.604	0.81 (0.37—1.78)
$152,000-$216,600	0.29	0.687	0.88 (0.46—1.68)	0.32	0.538	0.78 (0.35—1.74)
$216,700 and more	ref			ref		
***Parent's Age (yrs.) at Child's Birth***						
14 to 22	0.43	0.235	1.43 (0.79—2.58)	0.43	0.509	1.25 (0.64—2.45)
23 to 30	ref			ref		
31 to 40	0.11	0.146	0.83 (0.64—1.07)	0.13	0.224	0.83 (0.62—1.12)
41 to 54	0.22	0.126	0.53 (0.24—1.19)	0.41	0.699	0.82 (0.31—2.21)
***Household Size***						
Two	0.55	0.480	1.34 (0.60—3.00)	0.77	0.495	1.44 (0.51—4.09)
Three or Four	ref			ref		
Five or Six	0.13	0.848	0.98 (0.75—1.27)	0.14	0.615	0.93 (0.69—1.25)
***Immigration***						
Yes, in the last 10 years	0.37	0.053	1.58 (1.00—2.51)	^**^		
Yes, more than 10 years ago	0.21	0.893	0.97 (0.63—1.49)			
No, born in Canada	ref					
***Parent's Marital Status***						
Married/Common-Law	ref			ref		
Widowed/Separated/Divorced	0.14	**0.009**	0.39 (0.19—0.79)	0.18	**0.048**	0.49 (0.24—0.99)
Single/Never Married	0.23	0.604	0.87 (0.51—1.47)	0.24	0.550	0.84 (0.48—1.47)
***Parent's Sex***						
Male	ref			^**^		
Female	0.22	0.542	0.86 (0.52—1.41)			
***Parent's Education Level***						
High School or Less	0.11	**0.002**	0.53 (0.35—0.79)	0.11	**0.001**	0.49 (0.32—0.75)
Trade/College Certificate or Diploma	0.10	**0.004**	0.63 (0.46—0.86)	0.11	**0.011**	0.64 (0.45—0.90)
University, Undergraduate level	ref			ref		
University, Graduate level	0.19	0.690	0.92 (0.62—1.38)	0.27	0.991	1.00 (0.59—1.68)
***Aboriginal Identity of Child***						
Aboriginal Identity	0.23	0.462	0.81 (0.47—1.41)	0.32	0.842	0.93 (0.48—1.83)
Non-Aboriginal Identity	ref			ref		
***Region of Residence***						
Atlantic	ref			ref		
Quebec	0.28	**0.001**	1.69 (1.23—2.34)	0.39	**0.001**	1.95 (1.31—2.90)
Ontario	0.31	**0.002**	1.73 (1.22—2.46)	0.21	0.614	1.10 (0.76—1.60)
Prairies	0.14	0.836	1.03 (0.79—1.33)	0.10	**0.020**	0.72 (0.54—0.95)
British Columbia	0.16	0.912	0.98 (0.71—1.36)	0.11	**0.005**	0.61 (0.43—0.86)
Territories	0.17	0.282	0.80 (0.53—1.20)	0.17	0.158	0.72 (0.45—1.14)
***Parent Level of Vaccine KAB***						
Accurate Understanding	ref			ref		
Moderate Understanding	0.05	**0.000**	0.32 (0.23—0.44)	0.05	**0.000**	0.25 (0.18—0.36)
Poor Understanding	0.03	**0.000**	0.04 (0.01—0.16)	0.03	**0.000**	0.05 (0.02—0.14)
***Ever Decided to Delay a Vaccine(s)***						
Delayed Sometime	ref			ref		
Never Delayed	0.19	1.000	1.00 (0.69—1.46)	0.23	0.753	1.07 (0.70—1.64)
***Ever Refused Vaccine(s)***						
Refused Sometime	ref			ref		
Never Refused	0.18	0.912	1.02 (0.73—1.43)	0.24	0.286	1.23 (0.84—1.80)
***Ever Been Reluctant to Accept Vaccine(s)***						
Reluctant Sometime	ref			ref		
Never Reluctant	0.38	**0.000**	2.11 (1.49—2.99)	0.46	**0.000**	2.40 (1.65—3.51)

*n* = weighted sample; R2 = McFadden's R2 (likelihood ratio index), *SE*  Bootstrapped Standard Error; * = results with *p*-value < 0.05 are bold; *OR* Odds Ratio, *CI*  Confidence Interval; ^**^variable omitted from model for goodness of fit

Table representing predictors of sources that responding parents trust for vaccine information; results displayed as Standard Error, *p*-value and Odds Ratio with 95% Confidence Interval

^*^All data are weighted and bootstrapped to obtain estimates at the Canadian population level

**Table 7:** Predictors of Sources that Responding Parents Trust for Vaccine Information

	***Doctors***			***Nurses***		
	*n* = 290,000, R2 = 0.151			*n* = 270,000, R2 = 0.130		
	SE	p*	OR (95% CI)	SE	*p*	OR (95% CI)
***Total Household Income***						
$49,000 or less	0.21	0.103	0.52 (0.23—1.14)	0.21	0.125	0.57 (0.28—1.17)
$49,100-$98,000	0.18	0.053	0.49 (0.24—1.01)	0.28	0.592	0.83 (0.43—1.62)
$98,100-$151,900	0.26	0.321	0.69 (0.34—1.43)	0.32	0.841	0.94 (0.48—1.81)
$152,000-$216,600	0.25	0.243	0.63 (0.29—1.37)	0.32	0.732	0.89 (0.44—1.78)
$216,700 and more	ref			ref		
***Parent's Age (yrs.) at Child's Birth***						
14 to 22	0.36	0.905	1.04 (0.53—2.06)	0.34	0.994	1.00 (0.52—1.93)
23 to 30	ref			ref		
31 to 40	0.19	0.427	1.14 (0.82—1.58)	0.14	0.763	0.96 (0.72—1.27)
41 to 54	0.55	0.739	1.17 (0.47—2.93)	0.35	0.654	0.83 (0.36—1.90)
***Household Size***						
Two	0.46	0.903	0.94 (0.36—2.45)	0.51	0.836	1.10 (0.44—2.75)
Three or Four	ref			ref		
Five or Six	0.14	0.295	0.84 (0.61—1.16)	0.13	0.182	0.81 (0.59—1.10)
***Immigration***						
Yes, in the last 10 years	0.28	0.810	1.07 (0.64—1.79)	0.17	0.182	0.74 (0.48—1.15)
Yes, more than 10 years ago	0.19	0.348	0.80 (0.50—1.28)	0.11	**0.003**	0.54 (0.36—0.82)
No, born in Canada	ref			ref		
***Parent's Marital Status***						
Married/Common-Law	ref			ref		
Widowed/Separated/Divorced	0.31	0.599	0.82 (0.39—1.72)	0.39	0.896	0.95 (0.43—2.11)
Single/Never Married	0.23	0.352	0.75 (0.41—1.38)	0.19	0.133	0.63 (0.34—1.15)
***Parent's Sex***						
Male	^**^			^**^		
Female						
***Parent's Education Level***						
High School or Less	0.22	0.560	0.86 (0.53—1.41)	0.19	0.365	0.81 (0.51—1.28)
Trade/College Certificate or Diploma	0.20	0.886	1.03 (0.70—1.52)	0.17	0.481	0.88 (0.61—1.27)
University, Undergraduate level	ref			ref		
University, Graduate level	0.28	0.834	1.06 (0.63—1.78)	0.18	0.410	0.84 (0.55—1.28)
***Aboriginal Identity of Child***						
Aboriginal Identity	0.39	0.788	1.10 (0.55—2.19)	0.26	0.571	0.84 (0.46—1.53)
Non-Aboriginal Identity	ref			ref		
***Region of Residence***						
Atlantic	ref			ref		
Quebec	0.47	**0.002**	2.02 (1.28—3.17)	0.41	**0.013**	1.77 (1.13—2.79)
Ontario	0.25	0.541	1.14 (0.75—1.74)	0.14	0.260	0.82 (0.59—1.16)
Prairies	0.11	**0.027**	0.72 (0.54—0.96)	0.11	0.118	0.80 (0.61—1.06)
British Columbia	0.13	0.071	0.73 (0.51—1.03)	0.15	0.332	0.84 (0.60—1.19)
Territories	0.10	**0.001**	0.46 (0.29—0.71)	0.12	**0.006**	0.57 (0.38—0.85)
***Parent Level of Vaccine KAB***						
Very Good Understanding	ref			ref		
Moderate Understanding	0.05	**0.000**	0.27 (0.19—0.39)	0.06	**0.000**	0.33 (0.23—0.46)
Poor Understanding	0.03	**0.000**	0.05 (0.02—0.15)	0.03	**0.000**	0.06 (0.02—0.16)
***Ever Decided to Delay a Vaccine(s)***						
Delayed Sometime	ref			ref		
Never Delayed	0.26	0.542	1.15 (0.73—1.80)	0.26	0.226	1.28 (0.86—1.90)
***Ever Refused Vaccine(s)***						
Refused Sometime	ref			ref		
Never Refused	0.33	**0.015**	1.63 (1.10—2.42)	0.22	0.246	1.23 (0.87—1.75)
***Ever Been Reluctant to Accept Vaccine(s)***						
Reluctant Sometime	ref			ref		
Never Reluctant	0.54	**0.000**	2.69 (1.81—3.99)	0.40	**0.000**	2.03 (1.38—3.00)

*n* = weighted sample; R2 = McFadden's R2 (likelihood ratio index); *SE* Bootstrapped Standard Error; * = results with *p*-value < 0.05 are bold; *OR *Odds Ratio, *CI* Confidence Interval; ^**^variable omitted from model for goodness of fit

Table representing predictors of sources that responding parents trust for vaccine information; results displayed as Standard Error, *p*-value and Odds Ratio with 95% Confidence Interval

^*^All data are weighted and bootstrapped to obtain estimates at the Canadian population level

**Table 8:** Predictors of sources that responding parents trust for vaccine information

	***Pharmacists***			***Alt. Health Providers***		
	*n* = 250,000, R2 = 0.0932			*n* = 79,000, R2 = 0.0684		
	SE	p*	OR (95% CI)	SE	*p*	OR (95% CI)
***Total Household Income***						
$49,000 or less	0.22	0.237	0.69 (0.37—1.28)	0.93	**0.006**	2.64 (1.32—5.27)
$49,100-$98,000	0.24	0.494	0.82 (0.47—1.44)	0.71	**0.016**	2.18 (1.15—4.12)
$98,100-$151,900	0.26	0.691	0.89 (0.51—1.57)	0.52	0.096	1.68 (0.91—3.09)
$152,000-$216,600	0.31	0.991	1.00 (0.54—1.83)	0.68	**0.043**	2.00 (1.02—3.91)
$216,700 and more	ref			ref		
***Parent's Age (yrs.) at Child's Birth***						
14 to 22	0.28	0.981	0.99 (0.57—1.72)	0.27	0.794	0.93 (0.52—1.65)
23 to 30	ref			ref		
31 to 40	0.15	0.250	1.16 (0.90—1.48)	0.13	0.983	1.00 (0.77—1.29)
41 to 54	0.50	0.536	1.27 (0.59—2.75)	0.42	0.792	1.11 (0.52—2.33)
***Household Size***						
Two	0.59	0.574	1.29 (0.53—3.17)	0.64	0.375	1.47 (0.63—3.47)
Three or Four	ref			ref		
Five or Six	0.13	0.850	0.98 (0.75—1.27)	0.13	0.451	0.90 (0.68—1.19)
***Immigration***						
Yes, in the last 10 years	0.11	**0.002**	0.55 (0.38—0.80)	0.27	0.098	1.39 (0.94—2.05)
Yes, more than 10 years ago	0.09	**0.000**	0.47 (0.33—0.68)	0.17	0.315	0.81 (0.53—1.22)
No, born in Canada	ref			ref		
***Parent's Marital Status***						
Married/Common-Law	ref			ref		
Widowed/Separated/Divorced	0.45	0.435	1.31 (0.67—2.56)	0.18	0.054	0.50 (0.25—1.01)
Single/Never Married	0.32	0.918	1.03 (0.57—1.89)	0.28	0.855	1.05 (0.62—1.78)
***Parent's Sex***						
Male	ref			ref		
Female	0.27	0.198	1.30 (0.87—1.94)	0.40	**0.018**	1.73 (1.10—2.71)
***Parent's Education Level***						
High School or Less	0.14	0.159	0.77 (0.54—1.11)	0.25	0.237	1.27 (0.86—1.88)
Trade/College Certificate or Diploma	0.10	**0.020**	0.71 (0.54—0.95)	0.23	**0.002**	1.58 (1.18—2.11)
University, Undergraduate level	ref			ref		
University, Graduate level	0.14	0.184	0.79 (0.55—1.12)	0.18	0.303	0.80 (0.52—1.23)
***Aboriginal Identity of Child***						
Aboriginal Identity	0.23	0.541	0.85 (0.50—1.44)	0.30	0.681	1.12 (0.66—1.88)
Non-Aboriginal Identity	ref			ref		
***Region of Residence***						
Atlantic	ref			ref		
Quebec	0.36	**0.000**	2.00 (1.41—2.86)	0.18	0.275	1.18 (0.88—1.60)
Ontario	0.13	0.090	0.76 (0.55—1.05)	0.14	0.201	0.80 (0.56—1.13)
Prairies	0.12	0.160	0.82 (0.63—1.08)	0.14	0.742	1.04 (0.81—1.34)
British Columbia	0.11	**0.006**	0.63 (0.45—0.88)	0.16	0.801	0.96 (0.69—1.33)
Territories	0.13	**0.022**	0.63 (0.43—0.94)	0.22	0.830	1.05 (0.70—1.57)
***Parent Level of Vaccine KAB***						
Very Good Understanding	ref			ref		
Moderate Understanding	0.09	**0.000**	0.55 (0.39—0.76)	0.40	**0.000**	2.40 (1.73—3.34)
Poor Understanding	0.09	**0.000**	0.18 (0.07—0.47)	2.38	**0.000**	5.70 (2.51—12.95)
***Ever Decided to Delay a Vaccine(s)***						
Delayed Sometime	ref			ref		
Never Delayed	0.31	**0.002**	1.72 (1.21—2.44)	0.22	0.665	1.09 (0.74—1.61)
***Ever Refused Vaccine(s)***						
Refused Sometime	ref			ref		
Never Refused	0.17	0.778	1.05 (0.76—1.44)	0.15	0.707	0.94 (0.69—1.28)
***Ever Been Reluctant to Accept Vaccine(s)***						
Reluctant Sometime	ref			ref		
Never Reluctant	0.36	**0.000**	2.01 (1.41—2.86)	0.22	0.267	1.22 (0.86—1.73)

*n* = weighted sample; R2 = McFadden's R2 (likelihood ratio index); SE = Bootstrapped Standard Error; * = results with *p*-value < 0.05 are bold; *OR*  Odds Ratio, *CI*  Confidence Interval

Table representing predictors of sources that responding parents trust for vaccine information; results displayed as Standard Error, p-value and Odds Ratio with 95% Confidence Interval

^*^All data are weighted and bootstrapped to obtain estimates at the Canadian population level

**Table 9:** Predictors of sources that responding parents trust for vaccine information

	***Family***			***Friends***		
	*n* = 120,000, R2 = 0.0347			*n* = 82,000, R2 = 0.0404		
	SE	p*	OR (95% CI)	SE	*p*	OR (95% CI)
***Total Household Income***						
$49,000 or less	0.51	**0.028**	1.84 (1.07—3.15)	0.50	0.105	1.64 (0.90—2.97)
$49,100-$98,000	0.36	0.203	1.40 (0.84—2.33)	0.38	0.385	1.29 (0.73—2.29)
$98,100-$151,900	0.31	0.549	1.17 (0.70—1.96)	0.31	0.650	1.13 (0.66—1.95)
$152,000-$216,600	0.30	0.849	1.06 (0.61—1.83)	0.31	0.981	1.01 (0.55—1.85)
$216,700 and more	ref			ref		
***Parent's Age (yrs.) at Child's Birth***						
14 to 22	0.19	0.267	0.76 (0.46—1.24)	0.20	0.151	0.65 (0.36—1.17)
23 to 30	ref			ref		
31 to 40	0.15	**0.020**	1.31 (1.04—1.65)	0.14	0.334	1.13 (0.88—1.44)
41 to 54	0.81	**0.006**	2.47 (1.30—4.69)	0.42	0.767	1.12 (0.53—2.35)
***Household Size***						
Two	0.24	0.256	0.67 (0.33—1.34)	0.30	0.585	0.82 (0.39—1.69)
Three or Four	ref			ref		
Five or Six	0.12	0.792	0.97 (0.77—1.22)	0.11	0.315	0.88 (0.68—1.13)
***Immigration***						
Yes, in the last 10 years	0.22	0.122	1.30 (0.93—1.81)	0.20	0.606	1.10 (0.77—1.57)
Yes, more than 10 years ago	0.16	0.742	0.95 (0.68—1.32)	0.14	**0.044**	0.65 (0.42—0.99)
No, born in Canada	ref			ref		
***Parent's Marital Status***						
Married/Common-Law	ref			ref		
Widowed/Separated/Divorced	0.37	0.655	1.15 (0.62—2.16)	0.40	0.413	1.29 (0.70—2.36)
Single/Never Married	0.28	0.468	1.19 (0.75—1.89)	0.30	0.531	1.17 (0.71—1.94)
***Parent's Sex***						
Male	ref			ref		
Female	0.21	0.607	1.10 (0.76—1.59)	0.19	0.620	0.90 (0.59—1.37)
***Parent's Education Level***						
High School or Less	0.24	0.054	1.39 (0.99—1.94)	0.17	0.669	0.92 (0.64—1.33)
Trade/College Certificate or Diploma	0.17	**0.039**	1.31 (1.01—1.69)	0.17	0.144	1.23 (0.93—1.61)
University, Undergraduate level	ref			ref		
University, Graduate level	0.15	0.560	0.91 (0.66—1.25)	0.18	0.874	0.97 (0.68—1.38)
***Aboriginal Identity of Child***						
Aboriginal Identity	0.19	0.340	0.80 (0.50—1.27)	0.21	0.322	0.76 (0.44—1.32)
Non-Aboriginal Identity	ref			ref		
***Region of Residence***						
Atlantic	ref			ref		
Quebec	0.16	0.131	1.23 (0.94—1.59)	0.22	**0.000**	1.63 (1.26—2.12)
Ontario	0.11	**0.025**	0.72 (0.54—0.96)	0.11	**0.006**	0.64 (0.46—0.88)
Prairies	0.11	0.840	0.98 (0.79—1.21)	0.11	0.459	0.92 (0.73—1.16)
British Columbia	0.14	0.675	0.94 (0.71—1.25)	0.16	0.742	1.05 (0.79—1.41)
Territories	0.23	**0.016**	1.46 (1.07—1.99)	0.24	0.079	1.36 (0.97—1.91)
***Parent Level of Vaccine KAB***						
Very Good Understanding	ref			ref		
Moderate Understanding	0.21	0.208	1.24 (0.89—1.74)	0.19	0.559	1.11 (0.79—1.54)
Poor Understanding	0.43	0.928	1.04 (0.46—2.34)	0.80	0.138	1.88 (0.82—4.33)
***Ever Decided to Delay a Vaccine(s)***						
Delayed Sometime	ref			ref		
Never Delayed	0.29	**0.009**	1.61 (1.13—2.30)	0.32	**0.032**	1.56 (1.04—2.33)
***Ever Refused Vaccine(s)***						
Refused Sometime	ref			ref		
Never Refused	0.23	**0.022**	1.43 (1.05—1.95)	0.19	0.323	1.17 (0.85—1.62)
***Ever Been Reluctant to Accept Vaccine(s)***						
Reluctant Sometime	ref			ref		
Never Reluctant	0.20	0.291	1.20 (0.86—1.67)	0.30	0.058	1.48 (0.99—2.21)

n = weighted sample; R2 = McFadden's R2 (likelihood ratio index); SE = Bootstrapped Standard Error; * = results with *p*-value < 0.05 are bold; *OR* Odds Ratio, *CI* Confidence Interval

Table representing predictors of sources that responding parents trust for vaccine information; results displayed as Standard Error, *p*-value and Odds Ratio with 95% Confidence Interval

^*^All data are weighted and bootstrapped to obtain estimates at the Canadian population level

**Table 10:** Predictors of sources that responding parents trust for vaccine information

	***Prov. Min. of Health***			***Health Canada/PHAC***		
	*n* = 260,000, R2 = 0.116			*n* = 280,000, R2 = 0.145		
	SE	p*	OR (95% CI)	SE	*p*	OR (95% CI)
***Total Household Income***						
$49,000 or less	0.39	0.726	1.13 (0.57—2.23)	0.35	0.670	0.84 (0.37—1.91)
$49,100-$98,000	0.25	0.444	0.78 (0.42—1.46)	0.27	0.359	0.70 (0.33—1.50)
$98,100-$151,900	0.27	0.646	0.87 (0.47—1.60)	0.33	0.604	0.81 (0.37—1.78)
$152,000-$216,600	0.29	0.687	0.88 (0.46—1.68)	0.32	0.538	0.78 (0.35—1.74)
$216,700 and more	ref			ref		
***Parent's Age (yrs.) at Child's Birth***						
14 to 22	0.43	0.235	1.43 (0.79—2.58)	0.43	0.509	1.25 (0.64—2.45)
23 to 30	ref			ref		
31 to 40	0.11	0.146	0.83 (0.64—1.07)	0.13	0.224	0.83 (0.62—1.12)
41 to 54	0.22	0.126	0.53 (0.24—1.19)	0.41	0.699	0.82 (0.31—2.21)
***Household Size***						
Two	0.55	0.480	1.34 (0.60—3.00)	0.77	0.495	1.44 (0.51—4.09)
Three or Four	ref			ref		
Five or Six	0.13	0.848	0.98 (0.75—1.27)	0.14	0.615	0.93 (0.69—1.25)
***Immigration***						
Yes, in the last 10 years	0.37	0.053	1.58 (1.00—2.51)	**		
Yes, more than 10 years ago	0.21	0.893	0.97 (0.63—1.49)			
No, born in Canada	ref					
***Parent's Marital Status***						
Married/Common-Law	ref			ref		
Widowed/Separated/Divorced	0.14	**0.009**	0.39 (0.19—0.79)	0.18	**0.048**	0.49 (0.24—0.99)
Single/Never Married	0.23	0.604	0.87 (0.51—1.47)	0.24	0.550	0.84 (0.48—1.47)
***Parent's Sex***						
Male	ref			**		
Female	0.22	0.542	0.86 (0.52—1.41)			
***Parent's Education Level***						
High School or Less	0.11	**0.002**	0.53 (0.35—0.79)	0.11	**0.001**	0.49 (0.32—0.75)
Trade/College Certificate or Diploma	0.10	**0.004**	0.63 (0.46—0.86)	0.11	**0.011**	0.64 (0.45—0.90)
University, Undergraduate level	ref			ref		
University, Graduate level	0.19	0.690	0.92 (0.62—1.38)	0.27	0.991	1.00 (0.59—1.68)
***Aboriginal Identity of Child***						
Aboriginal Identity	0.23	0.462	0.81 (0.47—1.41)	0.32	0.842	0.93 (0.48—1.83)
Non-Aboriginal Identity	ref			ref		
***Region of Residence***						
Atlantic	ref			ref		
Quebec	0.28	**0.001**	1.69 (1.23—2.34)	0.39	**0.001**	1.95 (1.31—2.90)
Ontario	0.31	**0.002**	1.73 (1.22—2.46)	0.21	0.614	1.10 (0.76—1.60)
Prairies	0.14	0.836	1.03 (0.79—1.33)	0.10	**0.020**	0.72 (0.54—0.95)
British Columbia	0.16	0.912	0.98 (0.71—1.36)	0.11	**0.005**	0.61 (0.43—0.86)
Territories	0.17	0.282	0.80 (0.53—1.20)	0.17	0.158	0.72 (0.45—1.14)
***Parent Level of Vaccine KAB***						
Very Good Understanding	ref			ref		
Moderate Understanding	0.05	**0.000**	0.32 (0.23—0.44)	0.05	**0.000**	0.25 (0.18—0.36)
Poor Understanding	0.03	**0.000**	0.04 (0.01—0.16)	0.03	**0.000**	0.05 (0.02—0.14)
***Ever Decided to Delay a Vaccine(s)***						
Delayed Sometime	ref			ref		
Never Delayed	0.19	1.000	1.00 (0.69—1.46)	0.23	0.753	1.07 (0.70—1.64)
***Ever Refused Vaccine(s)***						
Refused Sometime	ref			ref		
Never Refused	0.18	0.912	1.02 (0.73—1.43)	0.24	0.286	1.23 (0.84—1.80)
***Ever Been Reluctant to Accept Vaccine(s)***						
Reluctant Sometime	ref			ref		
Never Reluctant	0.38	**0.000**	2.11 (1.49—2.99)	0.46	**0.000**	2.40 (1.65—3.51)

*n* = weighted sample; R2 = McFadden's R2 (likelihood ratio index); SE = Bootstrapped Standard Error; * = results with *p*-value < 0.05 are bold; *OR* Odds Ratio, *CI *Confidence Interval; **variable omitted from model for goodness of fit

Table representing predictors of sources that responding parents trust for vaccine information; results displayed as Standard Error, *p*-value and Odds Ratio with 95% Confidence Interval

^*^All data are weighted and bootstrapped to obtain estimates at the Canadian population level

### Doctors

A significantly higher proportion of parents who trusted doctors for vaccine information were from households with income greater than $98,100 (F = 8.84, *p* < 0.001), were 23 years old or older (F = 4.58, *p* = 0.003), in households of three to six people (F = 8.32, *p* < 0.001), either married/common law or widowed/separated/divorced (F = 20.21, *p* < 0.001), university educated (F = 10.45, *p* < 0.001), and identify their children as non-Indigenous (F = 10.97, *p* = 0.001). There was also a higher proportion of parents from Ontario, Quebec and the Atlantic provinces who trusted doctors compared to parents from the Prairies, British Columbia (BC), and the Territories (F = 6.75, *p* < 0.001). A significantly higher proportion of parents who trusted doctors for vaccine information who were found to have accurate or moderate vaccine KAB (F = 107.53, *p* < 0.001), and had never delayed (F = 23.38, *p* < 0.001), refused (F = 36.52, *p* < 0.001) or been reluctant to accept (F = 106.46, *p* < 0.001) a vaccine for their 2-year-old child.

The regression model showed that parents from Quebec were 2.02 times (95% CI 1.28–3.17) more likely to trust doctors for vaccine information than those from the Atlantic provinces, while parents from the Prairies (OR 0.72, 95% CI 0.54–0.96) and the Territories (OR 0.46, 95% CI 0.29–0.71) were less likely. Parents who were found to have a moderate (OR 0.27, 95% CI 0.19–0.39) or poor understanding of vaccine KAB (OR 0.05, 95% CI 0.02–0.15) were significantly less likely to trust doctors for vaccine information than those with accurate vaccine KAB. Parents who had never refused or been reluctant to accept a vaccine for their child were 1.63 times (95% CI 1.10–2.42) and 2.69 times (95% CI 1.81–3.99) more likely to trust doctors for vaccine information, respectively, than those who had.

### Nurses

A significantly higher proportion of parents with a household income > $49,000 trusted nurses for vaccine information (F = 14.54, *p* < 0.001). Additionally, parents who were 23–40 years old were more trusting (F = 3.59, *p* = 0.014) as were parents from households of three to six people (F = 10.38, *p* < 0.001), those who were born in Canada (F = 12.79,* p* < 0.001), married/common law or widowed/separated/divorced (F = 20.54, *p* < 0.001), those with university education (F = 7.34, *p* < 0.001), and those who identify their children as non-Indigenous (F = 6.98, *p* = 0.008). Provincially, the highest proportion of parents who trusted nurses was from Quebec and the lowest was from the Territories (F = 11.59, *p* < 0.001). Parents found to have accurate or moderate vaccine KAB (F = 87.56, *p* < 0.001), and those who had never delayed (F = 19.18, *p* < 0.001), refused (F = 10.2, *p* = 0.001), or been reluctant to accept a vaccine(s) (F = 52.35, *p* < 0.001) also trusted nurses more.

The regression model showed that parents who had immigrated to Canada more than 10 years ago (2006 or earlier) were less likely (OR 0.54, 95% CI 0.36–0.82) to trust nurses for vaccine information than parents who were born in Canada. Parents from the Territories were also less likely (OR 0.57, 95% CI 0.38—0.85) but those from Quebec were 1.77 times (95% CI 1.13—2.79) more likely to trust nurses than parents from the Atlantic provinces. Parents who were found to have a moderate (OR 0.33, 95% CI 0.23—0.46) or poor (OR 0.06, 95% CI 0.02—0.16) understanding of vaccine KAB were less likely to trust nurses for vaccine information than parents with an accurate understanding. Parents who had never been reluctant were 2.03 times (95% CI 1.38—3.00) more likely to trust nurses for vaccine information than those who had, but there was no predictive relationship for parents who had delayed or refused a vaccine.

### Pharmacists

A significantly higher proportion of parents from households with income > $49,000 trusted pharmacists (F = 6.82, *p* < 0.001), as well as those from a three or four-person household (F = 3.07, *p* = 0.047), married/common law (F = 3.06, *p* = 0.047), born in Canada (F = 16.44, *p* < 0.001), or had a university undergraduate or graduate education (F = 3.98, *p* = 0.008). Parents trusted pharmacists more if they were from Quebec or the Atlantic provinces and the least if they were from Ontario, BC and the Territories (F = 22.76, *p* < 0.001). There was also a significantly higher proportion of parents who trusted pharmacists who had been found to have accurate or moderate KAB (F = 34.88, *p* < 0.001), or if they had never delayed (F = 19.4, *p* < 0.001), refused (F = 4.0, *p* = 0.045) or been reluctant to accept a vaccine (F = 34.63, *p* < 0.001).

The regression model showed that parents who had immigrated to Canada in the last 10 years (OR 0.55, 95% CI 0.38—0.80) or more than 10 years ago (OR 0.47, 95% CI 0.33—0.68) were less likely to trust pharmacists than parents born in Canada. Parents with a trade/college certificate/diploma education (OR 0.71, 95% CI 0.54—0.95) were less likely to trust pharmacists for vaccine information than parents with undergraduate education. Compared to parents from the Atlantic provinces, parents from Quebec were 2.00 times (95% CI 1.41—2.86) more likely to trust pharmacists for vaccine information, whereas parents from BC (OR 0.63, 95% CI 0.45—0.88) and the Territories (OR 0.63, 95% CI 0.43—0.94) were less likely. Parents who had a moderate (OR 0.55, 95% CI 0.39—0.76) or poor (OR 0.18, 95% CI 0.07—0.47) understanding of vaccine KAB were less likely to trust pharmacists for vaccine information than parents with an accurate understanding. Parents who had never been reluctant or delayed a vaccine were 2.01 (95% CI 1.41—2.86) and 1.72 times (95% CI 1.21—2.44), respectively, more likely to trust pharmacists for vaccine information than those who had, but there was no significant association for parents who had refused a vaccine.

### Complementary and alternative medicine health providers

A significantly higher proportion of parents with household income < $49,000 (F = 16.65, *p* < 0.001) trusted CAM HCPs for vaccine information as well as parents who were 14–22 years old (F = 3.79, *p* = 0.011), parents from a two-person household (F = 3.11, *p* = 0.045), those who had immigrated to Canada in the last 10 years (F = 6.24, *p* = 0.002), had less than university education (F = 9.16, *p* < 0.001) or identify their child as Indigenous (F = 7.2, *p* = 0.007). A higher proportion of parents from the Territories or Quebec (F = 2.76, *p* = 0.046) and parents found to have poor vaccine KAB (F = 37.35, *p* < 0.001) trusted CAM HCPs. There were no associations between previously delaying, refusing, or being reluctant to accept a vaccine and trust in CAM HCPs.

The regression model found that parents with household income < $49,000, $49,100-$98,000 and $152,000-$216,600 were 2.64 (95% CI 1.32—5.27), 2.18 (95% CI 1.15—4.12), and 2.00 (95% CI 1.02—3.91) times, respectively, more likely to trust CAM HCPs for vaccine information than parents with income $216,700 or more. Parents with a trade/college certificate/diploma education were 1.58 (95% CI 1.18—2.11) times more likely to trust CAM HCPs than parents with an undergraduate education, as were female parents (OR 1.73, 95% CI 1.10—2.71) than male parents. Parents found to have a moderate (OR 2.40, 95% CI 1.73—3.34) or poor understanding (OR 5.70, 95% CI 2.51—12.95) of vaccine KAB were also more likely to trust CAM HCPs than parents with accurate KAB.

### Family

A significantly higher proportion of parents trusted family for vaccine information if they were from households with income < $98,000 (F = 8.73, *p* < 0.001), had immigrated to Canada (F = 7.30, *p* = 0.001), or had high school or trade/college education (F = 4.45, *p* = 0.004). A higher proportion of parents from the Territories trusted family for vaccine information than parents from other provinces (F = 4.85, *p* = 0.003) as did parents who had never delayed (F = 9.89, *p* = 0.002) or never refused a vaccine (F = 11.29, *p* = 0.001).

The regression model showed that parents that had a household income < $49,000 (OR 1.84, 95% CI 1.07—3.15) were more likely to trust family for vaccine information than parents with an income $216,700 or more. Parents were also more likely to trust family for vaccine information if they or were 31–40 years old (OR 1.31, 95% CI 1.04—1.65) or 41–54 years old (OR 2.47, 95% CI 1.30—4.69) when their child was born compared to parents who were 23–30 when their child was born. Parents were also more likely to trust family for vaccine information if they were from the Territories (OR 1.46, 95% CI 1.07—1.99), and less likely if they were from Ontario (OR 0.72, 95% CI 0.54—0.96) than parents from the Atlantic provinces. Parents who had never refused or delayed a vaccine were 1.43 times (95% CI 1.05—1.95) and 1.61 times (95% CI 1.13—2.30) more likely to trust family for vaccine information, respectively, than those who had.

## Friends

A significantly higher proportion of parents who trusted friends for vaccine information had a household income < $49,000 (F = 2.94, *p* = 0.020) or had immigrated to Canada in the last ten years (F = 3.4, *p* = 0.034). Provincially, a higher proportion of parents from the Territories and Quebec trusted friends for vaccine information and the smallest proportion was from Ontario (F = 15.16, *p* < 0.001). There was no association between trusting friends and vaccine KAB, but a higher proportion of parents who had never delayed a vaccine (F = 6.69, *p* = 0.010) or been reluctant (F = 3.84, *p* = 0.050) trusted friends.

The regression model showed that, compared to parents from the Atlantic provinces, parents were 1.63 times (95% CI 1.26—2.12) more likely to trust friends for vaccine information if they were from Quebec while parents from Ontario (OR 0.64, 95% CI 0.46—0.88) were less likely. Parents who had immigrated more than 10 years ago (OR 0.65, 95% CI 0.42—0.99) were less likely to trust friends for vaccine information than parents who were born in Canada. Parents who had never delayed a vaccine were 1.56 times (95% CI 1.04—2.33) more likely to trust friends for vaccine information than those who had, but there was no predictive relationship for refusal or reluctance.

### Provincial ministry of health

A significantly higher proportion of parents who trusted the PMH for vaccine information were from households with income > $98,100 (F = 2.93, *p* = 0.020), three to six-person households (F = 4.12, *p* = 0.016), had immigrated in the last 10 years (F = 4.43, *p* = 0.012) were married/common law (F = 5.53, *p* = 0.004), had a university education (F = 14.17, *p* < 0.001) and identify their children as non-Indigenous (F = 7.56, *p* = 0.006). The proportion of parents who trusted the PMH was the largest from Quebec and Ontario and smallest from the Territories (F = 3.202, *p* = 0.023). A higher proportion of parents found to have accurate or moderate vaccine KAB trusted the PMH (F = 57.8, *p* < 0.001), as well as those who had never delayed (F = 20.05, *p* < 0.001), refused (F = 11.81, *p* = 0.001) or been reluctant to accept (F = 86.43, *p* < 0.001) a vaccine in the past.

The regression model showed that parents who were widowed, separated, or divorced were less likely (OR 0.39, 95% CI 0.19—0.79) to trust the PMH for vaccine information than parents who were married/common law, as were parents with high school education or less (OR 0.53, 95% CI 0.35—0.79) or trade/college certificate/diploma (OR 0.63, 95% CI 0.46—0.86) than those with an undergraduate education. Parents were 1.69 times (95% CI 1.23—2.34) and 1.73 times (95% CI 1.22—2.46), respectively, more likely to trust the PMH for vaccine information if they were from Quebec or Ontario than if they were from the Atlantic provinces. Parents were less likely to trust the PMH if they were found to have moderate (OR 0.32, 95% CI 0.23—0.44) or poor (OR 0.04, 95% CI 0.01—0.16) vaccine KAB than if they had accurate KAB. Parents were 2.11 times (95% CI 1.49—2.99) more likely to trust the PHM if they had never been reluctant to accept a vaccine for their child but there was no significant relationship for refusing or delaying a vaccine.

### Health Canada/Public health agency of Canada

A significantly higher proportion of parents with a household income > $98,100 trusted HC/PHAC for vaccine information (F = 4.78, *p* = 0.001) as did parents from a three- or four-person household (F = 4.66, *p* = 0.010), those who had immigrated in the last 10 years (F = 4.25, *p* = 0.015), were married/common law (F = 4.79, *p* = 0.008), had a university education (F = 14.24, *p* < 0.001) or identify their child as non-Indigenous (F = 6.19, *p* = 0.013). The proportion of parents who trusted HC/PHAC was largest if they were from Quebec and smallest if they were from the Territories (F = 6.27, *p* < 0.001). A significantly higher proportion of parents who trusted HC/PHAC were found to have accurate or moderate vaccine KAB (F = 94.37, *p* < 0.001), or had never delayed (F = 30.78, *p* < 0.001), refused (F = 14.31, *p* < 0.001), or been reluctant to accept a vaccine (F = 86.44, *p* < 0.001) in the past.

The regression model showed that parents who were widowed, separated, or divorced were less likely (OR 0.49, 95% CI 0.24—0.99) to trust HC/PHAC for vaccine information than parents who were married/common law, as were parents with high school or less (OR 0.49, 95% CI 0.32—0.75), or trade/college certificate/diploma education (OR 0.64, 95% CI 0.45—0.90) compared to parents with an undergraduate education. Compared to parents from the Atlantic provinces, parents were 1.95 times (95% CI 1.31—2.90) more likely to trust HC/PHAC if they were from Quebec but parents from the Prairies (OR 0.72, 95% CI 0.54—0.95) or BC (OR 0.61, 95% CI 0.43—0.86) were less likely. Parents were also less likely to trust HC/PHAC if they had moderate (OR 0.25, 95% CI 0.18—0.36) or poor (OR 0.05, 95% CI 0.02—0.14) vaccine KAB than parents with accurate KAB. Parents who had never been reluctant to accept a vaccine were 2.40 times (95% CI 1.65—3.51) more likely to trust HC/PHAC than if they had, but there was no predictive relationship for parents who had refused or delayed a vaccine.

## Discussion

This study provides further understanding about sources that parents of 2-year-old children who have received at least one vaccine trust for vaccine information by showing that 83.4% of parents trust doctors for vaccine information, 70.6–80.9% trust pharmacists, PMH, nurses and HC/PHAC, 34.4% trust family and 23% trust friends and CAM HCPs. Similarly, data from the United States found that parents trusted doctors for vaccine-safety information most often (76%) followed by other HCP (26%), government vaccine experts/officials (23%), family and friends (15%) and celebrities (2%) [30] although trust in provincial and federal government sources in Canada was nearly three times higher.

### Influence of trust on vaccine decisions

Parents who had never been reluctant were more than twice as likely to trust doctors, nurses, pharmacists, PMH and HC/PHAC than those who were reluctant. Parents who had never delayed a vaccine were more likely to trust pharmacists, family and friends, and parents who had never refused a vaccine for their 2-year-old child were more likely to trust doctors and family. This supports previous research that shows that parents who trust doctors, pharmacists and government are more likely to vaccinate [4–11, 16]. Parents who have never been reluctant appear to trust healthcare systems, HCPs, pharmaceutical companies and governments to have the necessary competence and good intentions to provide safe and effective vaccines for their children [1, 31].

### Influence of knowledge, attitudes and beliefs on trust

This study showed that parents who have poor or moderate vaccine KAB were significantly less likely to trust doctors, nurses, pharmacists, PMH and HC/PHAC. Studies show that parents who are “pro-vaccine” or “hesitant” are more likely to trust physicians than those who are “anti-vaccine” [32, 33] though this may be because parents choose physicians they trust [33] as parents who are “hesitant” may also trust HCPs less [34]. Poor vaccine KAB can result in decreased vaccine acceptance [6, 17, 19, 25]; however, parents who trust their vaccine provider may accept vaccines even if their knowledge about them is incomplete [5, 6, 21, 35].

### Knowledge, attitudes and beliefs & complementary and alternative medicine health care providers

Parents were more likely to trust CAM HCPs if they had moderate or poor vaccine KAB or if they had a trade/college education. This study also found that three income levels doubled the likelihood of trusting CAM HCPs for vaccine information: < $49,000; $49,100-$98,000; and, $152,000-$216,600. However, that difference didn’t necessarily translate into behaviour since there was no significant relationship in this study between trusting CAM HCPs and delaying, refusing or being reluctant to accept a vaccine. While this appears to contradict previous research that has found an association between CAM medicine and vaccine delay or refusal [5, 19, 20], this study excluded parents who had refused all vaccines for their two-year-old child and also does not specify which CAM HCPs parents are accessing and to what extent they inform their health decisions.

### Friends and family

Parents who trusted family for vaccine information were more likely to have never delayed or refused a vaccine for their child. They were also more likely to be from households with income < $49,000, 31 years or older when their child was born, and more likely to have a trade/college school education. Similarly, parents who trusted friends for vaccine information were more likely to have never delayed a vaccine for their child. Parents were less likely to trust friends if they had immigrated more than 10 years ago, but no other variables predicted trust in friends except regional differences. Other studies have found that trusting friends increased vaccine hesitancy [34, 36] or had no impact on vaccine hesitancy [32, 37]. Approximately 97% of Canadian children have received at least one vaccine, and given this high vaccine rate, it is unsurprising that family and friends may provide support for vaccination regardless of income, age or education level.

### Influence of demographics on trust

This study found important relationships between trust and province of residence, immigration history, education level, marital status, household size and Indigenous identity. The findings show that province of residence was an important predictor of trust in certain regions. For instance, parents from Quebec were more likely to trust doctors, nurses, pharmacists, friends, PMH and HC/PHAC while parents from Ontario were more likely to trust the PMH, but less likely to trust friends or family for vaccine information. Parents from the Prairies and BC were less likely to trust HC/PHAC. Parents from BC and the Territories were less likely to trust nurses and pharmacists. In addition, parents from the Territories were also less likely to trust doctors, but more likely to trust family. These regional differences are difficult to interpret without further information, but it is possible that differences across Canada in who provides vaccines, and the historical sense of alienation in western Canada [38] could contribute to the lack of trust in HC/PHAC shown in the Prairies and BC. The lack of trust in biomedical HCPs in the Territories is likely related to decreased access to consistent medical care due to geographical distance and population size, and negative current and historical experiences with healthcare by Indigenous peoples in northern Canada [39], which in turn may lead to increased reliance on family. However, trusting family does not predict vaccine delay or refusal, but rather may provide an opportunity to support community members as valuable vaccine resources.

Parents who immigrated to Canada more than 10 years ago were less likely to trust nurses, pharmacists or friends compared to parents who had been born in Canada, and parents who had immigrated within the last 10 years were less likely to trust pharmacists. Other research has shown that parents of 2-year-old children who have immigrated to Canada were more likely to have accepted all vaccines than those born in Canada with the strongest predictor being from a country with high vaccine coverage (i.e.: Southeast and Northeast Asia) [40], and trust in Canadian HCPs, vaccine research and regulation [41], This finding could be related to a cultural preference or comfort level with doctors providing vaccines information rather than nurses or pharmacists.

In this study, parents with high school or trade/college education were less likely to trust the PMH, HC/PHAC, and pharmacists, and more likely to trust CAM HCPs and family. Prior studies have not evaluated the association between education and trust. However, previous studies show that poorer education does not directly predict vaccine acceptance, but may predict lower vaccine coverage [25, 42], higher vaccine coverage [18, 43], or have no effect at all [17, 19]. Three of these studies asked KAB questions which could be considered a proxy for vaccine hesitancy, but they did not measure trust or specific vaccine decisions (i.e.: reluctance, delay or refusal). Vaccine providers should be aware of the potential for parents who have less education to be less trusting of government levels of health, and further research is needed to replicate and further investigate the impact of education on trust.

Parents who were widowed/separated/divorced were less likely to trust the PMH and HC/PHAC. Because the cohort included in this survey was parents of 2-year-old children, it is possible that parents who fall into this category experienced the loss of a partner or relationship changes since their child was born. Prior studies show that past life experiences and trauma impact trust and vaccine decisions [1, 44], but have not examined how the impact may differ between sources of vaccine information or how long these effects last. 

Household size was not a significant predictor of sources that parents trust for vaccine information. Prior research has shown that larger households predict vaccine delay or refusal [16, 18, 20]. The lack of significant predictors between household size and trust may lend support to the conjecture that vaccine delays are the result of complicated household schedules in larger families [45]. However, household size was significantly associated with trusting doctors, nurses, pharmacists, CAM HCPs, PMH, and HC/PHAC, and warrants further research to understand the impact of household size on whom parents trust and vaccine decisions.

Similarly, Indigenous identity was significantly associated with trust in doctors, nurses, CAM HCPs, family, PMH, and HC/PHAC but did not remain in the regression models. Research has shown that historical experiences impact trust, especially for marginalized groups [1] including Indigenous peoples in Canada [3] and therefore these results should be interpreted with caution.

### Limitations

This study has several limitations. First, the sample was drawn from parents receiving Child Health Benefits and excluded parents living on First Nations reserves or institutionalized children. According to 2016 census data, 4.9% of Canada’s total population identify as Indigenous; 60% of Indigenous people in Canada identify as First Nations, 36% identify as Métis, and 4% identify as Inuit [46]. 44% of First Nations people who have Registered or Treaty status (76% of First Nations) live on a reserve while Métis residents are more likely to live in other rural or urban communities and Inuit residents tend to live in Canada’s north [46]. Hence, our sample under-represents First Nations children in Canada. This study was also unable to analyse specific Indigenous identities (i.e., Métis, First Nations, or Inuit) due to small cell sizes and therefore unable to differentiate between the experiences of different Indigenous groups within Canada. Secondly, the variable used for KAB encompassed several different aspects of vaccine knowledge and may have resulted in an overgeneralized understanding of parents’ vaccine KAB. Third, trust was assessed using a single item-measure in terms of sources that parents trusted for vaccine information which omitted other aspects of vaccination including trust in developer and provider competence, historical experiences, and generalised trust in members of society [1]. Fourth, social media was not included in the trust assessment in this survey. This is information that may have provided important insights into whom parents trust for vaccine information since social media is a growing source of vaccine misinformation and confirmation bias [47, 48] with a negative impact on vaccine coverage [49]. Fifth, the survey description does not indicate how the responding parent or guardian was chosen. Finally, the cNICS 2017 did not include KAB data for parents who had refused all vaccines and therefore does not adequately represent this population.

## Conclusions

This study showed that parents with accurate vaccine KAB were never reluctant to vaccinate and trusted doctors, nurses, pharmacists, PMH and HC/PHAC. Parents who had never delayed a vaccine were more likely to trust pharmacists, family and friends, and those who had never refused were more likely to trust doctors and family. Trust was predicted by income, age, gender, province of residence, education level, marital status, immigration, vaccine decisions and KAB. Future studies are needed to understand which beliefs predict vaccine decisions, how parents develop their vaccine beliefs, and how they react to vaccine information [50] to develop strategies that address vaccine hesitancy in parents who have lower KAB scores.

## Data Availability

The data that support the findings of this study are available from Statistics Canada but restrictions apply to the availability of these data, which were used under license for the current study, and so are not publicly available. Data are however available from the authors upon reasonable request and with permission of Statistics Canada. To submit an approval request for this data, please contact Dr. Ruben Mercado (Statistics Canada) at sky.rdc@usask.ca.
